# Applying social entrepreneurship to One Health and climate education: a retrospective analysis of pre- and post-course survey data for a One Health and Global Food Security course

**DOI:** 10.3389/fvets.2026.1828852

**Published:** 2026-06-03

**Authors:** Brianna Parsons, Brittany Watson

**Affiliations:** 1 University of Pennsylvania School of Veterinary Medicine, Philadelphia, PA, United States; 2 FAIR Farms Gambia, Busura, Gambia

**Keywords:** climate, education, one health, operationalization, social entrepreneurship, sustainability, systems thinking

## Abstract

One Health is a concept of interconnectivity between human, animal, and environmental health, and also an approach to creating stronger health systems and outcomes for all. Teaching One Health as an approach is pedagogically challenging, as it requires both acquisition of new knowledge and the application of the acquired knowledge (the One Health approach) to be effective. Several core competencies have defined essential skills and behaviors to support One Health education, but more research is needed to fully operationalize the field. Social entrepreneurship is a systems-thinking approach that uses business methodologies to solve social challenges by blending financial revenue with social impacts. This approach may help provide an application-based paradigm to increase systems thinking in One Health education. This study aimed to evaluate how careful integration of social entrepreneurship into the One Health and Global Food Security course has supported students’ learning. An exploratory retrospective analysis was performed on participant-matched pre- and post-course surveys from undergraduate, graduate, and professional students enrolled in the course in 2024 (*n* = 11) and 2025 (*n* = 9). Students’ self-assessed knowledge of six key topics (such as food systems, sustainability, social entrepreneurship, One Health, global food security, and impact assessment) increased significantly (*p* < 0.001) through participation in the course. Students’ understanding of social entrepreneurship was evaluated by significant improvements (*p* < 0.001) in rubric-graded definitions of the term pre- and post-course, and students rated several aspects of applied social entrepreneurship (such as pitch presentation and case study project) as highly impactful to their learning. Additionally, students indicated they were likely to use social entrepreneurship in their future and to recommend social entrepreneurship as a tool for climate action. Taken together, these results suggest social entrepreneurship as a curricular approach may help support One Health operationalization and education.

## 1 Introduction

One Health is a unifying concept, recognizing the interconnectivity between humans, animals, and environmental health (1–3). As an approach, One Health builds solutions for stronger systems by mobilizing diverse, multisectoral teams with transdisciplinary perspectives to identify commonalities in structural causes of ill health and create new integrated and supportive systems. Both the concept of interconnectivity and the approach to building solutions define One Health, a unique field perceived by many as capable of solving society’s toughest challenges, such as the United Nations Sustainable Development Goals (1).

One Health higher education seeks to prepare tomorrow’s leaders to address societal “wicked” challenges through robust interdisciplinary content, transdisciplinary teams, systems thinking, and community-based collaborations (2, 4, 5). However, One Health education is complex and diverse, as it must adequately teach both the concept and the approach. The concept of human, animal and environmental health as interconnected encompasses several disciplines or topic areas–zoonotic disease, food systems, climate, antimicrobial resistance, health systems, and socioecological systems–and thus the delivery of One Health education can vary greatly across programs (2, 6, 7). Nonetheless, teaching One Health as an approach–how to conceptualize and solve complex modern social challenges–is more complicated pedagogically. It requires not only the acquisition of new content knowledge (such as what is a One Health approach) but also the application of that knowledge to build systematic solutions to a wide variety of societal challenges.

To create frameworks and standards for teaching One Health, several groups have identified core competencies, or the essential skills, behaviors, and attitudes for success that complement topic-specific and content-based coursework (8, 9). By examining findings from several working groups, initial One Health competencies were described across seven domains, including management, communication and informatics, values and ethics, leadership, teams and collaboration, roles and responsibilities, and systems thinking (9). Two years later, these competencies were reframed into three categories, including health knowledge; global and local issues in humans, animals, plants, and the environment; and professional characteristics (7). Another model proposed several skills, values and attitudes, and knowledge and awareness as competencies for One Health education (6), aligning with the definitions proposed by the OHHLEP. Several specific One Health competencies overlap with the professional competencies of health accrediting institutions, including the American Veterinary Medical Council on Education (AVMA COE), Association of American Veterinary Medical Colleges Competency-Based Veterinary Education, and Association of American Medical Colleges Liaison Committee on Medical Education (10). Despite differences and revisions over the years, fundamentally, there is agreement that One Health education and the approach itself requires professional and interpersonal skill development, interdisciplinarity, systems thinking, and inter-professional teambuilding (6, 7, 9, 11–13).

As a field, One Health has gained momentum, financing, credibility, and institutional support over the decades, especially following the coronavirus disease 19 (COVID-19) pandemic, which highlighted the direct links between humans, animals, and environmental health. The One Health approach was adopted by the Tripartite partnership of the World Health Organization (WHO), Food and Agricultural Organization of the United Nations (FAO), and the World Organization for Animal Health (WOAH) in 2010, with recent establishment of a quadripartite through the addition of the United Nations Environmental Program (UNEP) in 2022 ((14), 2021; (15–17)). OHHLEP supported the Quadripartite by defining a One Health Theory of Change describing a proposed approach, outcomes, pathways of change, and impact (1, 16). One Health educational programs and research networks have similarly expanded during this period (4).

As One Health experiences a moment of rapid growth, it is not without criticism. Contemporary criticisms, and even those dating back nearly a decade, describe One Health as a health profession driven concept largely focused on revising definitions and frameworks, with limited outcomes beyond empirical studies focused primarily on zoonotic disease and antimicrobial resistance, and without sufficient exploration of system connectivity, structural problems, or root causes of inequity (18–21). Some claim the increased political and financial attention to the field results in siloing or splintering of efforts (22). Together these critiques capture One Health operationalization, an identified challenge for which the Lancet One Health Commission proposed three levers to operationalize the One Health concept into action–governance, economics, and knowledge (2).

Social entrepreneurship is an operational approach to build new business models to solve social and environmental challenges by blending profit with social impact (23, 24). Social entrepreneurship and systems thinking are complementary, and at times co-taught approaches, comprising several specific frameworks and methods. Like the One Health approach, social entrepreneurship is increasingly lauded as relevant for solving social and environmental problems, Bansal et al. (25) as this human-centered approach incorporates various sources of information to create systems-based frameworks to understand and respond to complex, societal challenges (24, 26). Originating from entrepreneurship and business methodologies to de-risk ventures, social entrepreneurship is explicitly concerned with social problems and seeks blended financial and social returns to build new models for improving social conditions (23–27). While social entrepreneurship delivers actionability, it is criticized for having limited public accountability, as its business solutions to social problems create structures of change outside of the publicly accountable government sector (28). With many frameworks and approaches, such as Logic Models or Theory of Change and Lean Startup (29, 30), social entrepreneurship exercises may be customized to the objectives, goals, and needs of specific One Health courses or instructors, making the approach relevant for systems thinking within One Health.

To provide context on One Health operationalization through knowledge systems, this study sought to evaluate the incorporation of social entrepreneurship in a One Health and Global Food Security course. This course explores food systems content and integrates social entrepreneurship throughout as a systems-thinking framework. Students apply this approach through a semester-long case study project designed to create real-life solutions to existing food system challenges. As One Health educators, this study find social entrepreneurship relevant as a One Health approach and hypothesize that the careful integration of social entrepreneurship into this course may provide students with tools to solve complex One Health problems. This study retrospectively examines student learning outcomes from matched pre- and post-course surveys for the One Health and Global Food Security course at the University of Pennsylvania in 2024 (*n* = 11) and 2025 (*n* = 9). By examining student learning outcomes in this One Health course, the study aimed to understand principles for systems thinking and One Health education in order to help expand operational coursework for the One Health focused students.

## 2 Methodology

A retrospective analysis of student learning evaluation data from the Penn Vet One Health and Global Food Security course was conducted for 2024 and 2025. The University of Pennsylvania Institutional Review Board reviewed the study protocol and found it exempt (26–609). Enrolled students completed a pre- and post-course survey in Qualtrics consisting of several Likert scale questions to self-assess knowledge of, and the importance of, global health across six key topics (such as food systems, sustainability, social entrepreneurship, One Health, global food security, and impact assessment). Students were informed that survey answers would remain anonymous and were encouraged to respond honestly to help contribute to course improvement and development in subsequent years. The pre-course survey was administered at the beginning of the first class for ease of completion and so students could respond without any influence from starting the course. The post-course survey was completed on students’ own time after all classes and final pitch presentations were completed, allowing time for reflection. The full survey questionnaire with Likert scales is listed in Supplementary Table 1, and definitions of the six key topics on the survey are provided in Supplementary Table 2.

In addition to the previous Likert scale questions, the post-course survey included additional Likert scale questions to assess student learning, attitudes, and behaviors, asking students to reflect on how six course activities impacted their learning and how likely they were to engage in six future activities.

The 2024 and 2025 pre- and post-course survey data were compiled into one dataset. Using a deidentified participant identify (ID) number, pre- and post-answers were matched for each student. After coding, compiling, and cleaning up the data, analysis was performed in SPSS. Survey results for which pre- and post-course matching could not be performed (*n* = 2) were removed from the analysis.

For Likert scale questions, there has been longstanding debate about whether to treat Likert data as ordinal or interval-level (continuous) data (31, 32), and subsequently whether to analyze it using the non-parametric (33) or parametric tests (34). While the questions has been shown that parametric tests including the F-test, are robust to some violations of the interval data assumption and normality (34, 35), this study used the non-parametric tests, based on the small sample size, non-normality, and limited scope of the data, and to align with common practices in medical education evaluation (36–38).

For Likert scale questions asked in both pre- and post-course surveys (such as self-assessed knowledge and importance to global health), answers were converted to their numerical 5-point equivalent and descriptive statistics were run (including median, mean, range, standard deviation, and frequencies). To assess statistical significance, the Wilcoxon Signed Rank test was performed as a non-parametric analysis of related samples. Effect size (*r*) is also reported for each item, calculated using the following equation:

$r=\frac{Z}{\sqrt{N}}$

Where, *Z* = the standardized test statistic from the Wilcoxon Signed Rank test, and *N* = the total number of observations (such as the number of paired samples included in the analysis).

To assess multiplicity when evaluating several related factors, such as importance to global health, a Bonferroni correction was performed using the following formula to adjust *p*-value significance:

*α*_adjusted_ = $\frac{\alpha }{\text{number of tests}.}$

Where 𝛼 = the original significance threshold, and 𝛼_adjusted_ = the new, stricter threshold for significance after correction.

A composite score was created for each Likert item with a corresponding pre- and post-course answer (Composite score = Post–Pre) to create composite scores for six knowledge topics and six importance topics (such as food systems, sustainability, social entrepreneurship, One Health, global food security, and impact assessment). Descriptive statistics for each composite score were run (including median, mean, range, standard deviation, and frequencies).

Students were asked to define social entrepreneurship in the pre- and post-course survey. Each definition was scored on a rubric (listed in Supplementary Table 3) from 0 to 3 with the following code: 0: no answer in pre-course (did not know what the term was), 1: wrong definition, 2: definition includes either “business approach/methodology for social change” OR “blending social impact and financial revenue”, and 3: definition includes BOTH terms. Definition scoring was blinded to student ID, but pre- and post-course definition was known. Inter-rater reliability testing was performed with 84% accuracy, indicating a useful assessment rubric. A composite score was created for the definitions (Composite score = Post-course Rubric Score – Pre-course rubric score). Descriptive statistics (including median, mean, range, standard deviation, and frequencies) were run for the composite score. To assess statistical significance, Wilcoxon Signed Rank test and effect size (*r*) are performed.

Lastly, some Likert items, designed to assess student learning, attitudes, and behaviors were evaluated only in the post-course survey. These items were analyzed using descriptive statistics (including mean, median, range, standard deviation, and frequencies).

### 2.1 Educational design, learning environments, and course objectives

The One Health and Global Food Security course is an elective course open to undergraduate, graduate, and professional students. The course began in the early 2000s, co-taught and cross-listed through a veterinary school and an undergraduate anthropology department, and was run largely as an interdisciplinary survey course with several guest lecturers. Since its origin, the course has explored global food security, asking questions of how we feed the world in the face of population growth, inequality, and climate change. In 2021, a reorganization of course material focused on modernizing and balancing food system content around the One Health Approach. In subsequent course years, social entrepreneurship was integrated as a cross-cutting theme, with students required to apply these frameworks through a semester-long problem-based case study project. This study focuses on course evaluation data from 2024 and 2025 after these course changes were fully implemented.

The course organizer led topic-based lectures, “chunk & check” debriefs after guest lectures, and in-class learning activities on social entrepreneurship; organized guest lecturers; and provided student feedback and grades throughout the 15-week course. The course was held in 3-h sessions once a week from 27 August 27 to 3 December, 2024, and again from 26 August 26 to 2 December, 2025. Enrolled students represented several degrees, schools, and programs, as shown in Table 1.

**Table 1:** Schools and programs represented in 2024 and 2025 one health and global food security course student enrollment.

Year	Enrolled students	Programs represented
2024	12	Veterinary (10) Masters of Public Health (2)
2025	10	Veterinary (4) Veterinary-PhD dual-degree (1) Bachelor of Science in Engineering (1) Masters of Public Health (2) Masters of Business Administration (1) Masters of Public Administration (1)

Each year students received 25–28 guest lecturers on several topics related to Global Food Security and One Health. While the majority of guest lecturers spoke to both 2024 and 2025 classes, some lectures were only given to one class year based on instructor availability and opportunities to present engaging, relevant materials. To scaffold new content learning, guest lectures were organized according to a Sustainability Framework, defining sustainability as competing but overlapping factors of social, environmental, and economic sustainability, with trade-offs influenced and constrained by socio-economic and geo-political factors (39).

The major course assignment was a semester-long, 3-part case study project, in which students chose a global food security topic of interest, investigated its multifaceted challenges, and devised a relevant solution using social entrepreneurship. Additional course requirements included discussion posts on the required course book “Winners Take All: The Elite Charade of Changing the World” (28), discussion and reflection posts for selected guest lecturers, and attendance and participation in class activities. Course learning objectives are listed in Table 2.

**Table 2:** Learning objectives for the one health and global food security course.

Topic area	Learning objectives
Introduction to global food Security and sustainability	Describe intersecting global challenges (population growth, climate change, inequality) Describe concepts of systems-thinking and wicked problems Describe basic entrepreneurial principles (lean methods, impact assessment, human centered design) Define Sustainability and One Health
Animal production systems	Evaluate the role of livestock (poultry, dairy, beef, swine, and aquaculture) in sustainable food systems Describe the production efficiency concept Consider tradeoffs in how society produces animal proteins for human consumption
Introduction to cropping	Evaluate the role of cropping in sustainable food systems Describe concepts of plant breeding and genetics, precision agriculture, and GMO technology Consider tradeoffs in how society produces plant foods for human consumption.
Environmental issues in agriculture, conservation, and wildlife	Describe the metrics associated with animal agriculture, climate change and environmental impact Describe intensive and extensive farming, as well as land sparing and land sharing ecological approaches Apply concepts of systems thinking and entrepreneurship Interpret findings from scientific manuscripts across disciplines to draw conclusions
Food loss and food waste	Describe the drivers of food loss and food waste across the globe Evaluate the role of individual and systemic solutions to food loss and waste
Social issues in agriculture	Interpret the structural and systemic challenges facing indigenous communities in the U. S. Interpret the structural and systemic challenges resulting in unequal climate outcomes for different populations across the world Evaluate implicit biases and structural inequities to explore the role of individual action vs. structural reform Describe health disparities of rural communities, including causes and impacts Recognize the nutritional values of foods derived from livestock. Recall how various livestock production methods have different environmental impacts Evaluate trade-offs in food production (environmental vs. social)
Economic issues in agriculture	Describe principles of consolidation in the agricultural industry Describe the production efficiency concept Describe the food supply chain and understand importance of markets and access in food systems
Sociopolitical considerations in agriculture	Describe elements of public policy that affect global food security Describe elements of negotiations, sociopolitical strategy, and public policy as it relates to implementing effective initiatives Recognize differences in intention and impact
Social entrepreneurship as a cross-cutting theme	Apply concepts of systems thinking and social entrepreneurship Evaluate complex sustainability tradeoffs across One Health and Food System issues Create a real-life case study project throughout the semester developing a comprehensive solution to a food system challenge of choice, evaluating tradeoffs and stakeholder experiences, building a logic model/theory of change, and presenting the idea through a pitch presentation in the final class

Social entrepreneurship was embedded throughout the course as a cross-cutting theme to provide students with actionable, systems-thinking frameworks for social and One Health solution building. To deepen students’ understanding, the book “Winners Take All” (28) was required reading, with discussion board posts and in-class discussions used to explore advantages and drawbacks of this approach. The specific social entrepreneurship frameworks studied in this course are presented in Table 3.

**Table 3:** Social entrepreneurship methods used in the one health and global food security course.

Name of social entrepreneurship method	Use in class	Learning objective	Other comments
Introduction to social entrepreneurship	Lecture and discussion of various methodologies and approaches in Social Entrepreneurship	Students gain familiarity with a wide variety of methods at their disposal within social entrepreneurship	Many of these methodologies are revisited in subsequent class periods, including active learning activities Topics: *Entrepreneurship vs. Social Entrepreneurship; Equity in Entrepreneurship; Uncertainty & Risk; Opportunity recognition; Human-Centered Design; Lean Startup Model; Logic Models; Understanding stakeholder perspectives; addressing root causes*
Beneficiary-centered screen in	Active learning group activity during first class Key questions designed to interrogate core understandings of a particular problem, a proposed solution, and possible behavior changes by beneficiaries/ stakeholders in response to the solution Exercise includes screen-in criteria with questions and scoring to evaluate the potential positive social impact of a given solution	Students apply beneficiary-centered design by asking key questions on a selected ‘problem,’ a proposed solution, and considering behaviors changes in response to implementation of that solution	Students are given several example problems and proposed solutions and work in teams to evaluate the potential impact of the problem/solution by carefully considering the impact on those most affected by the problem
Framing and scoping a solution	Active learning group activity during first class A key principle in Social Entrepreneurship is ensuring a potential solution provides both social impact and financial revenue	Students understand and can apply framing and scoping of solutions to develop reasonable social enterprises	As a business minded approach to changemaking, social entrepreneurship considers financial sustainability as a component to overall sustainability. As such, a social enterprise generates revenue to offset/eliminate operating expenses or even generate profit to sustain and grown successful initiatives through financing controlled by the organization itself.
Problem trees	Active learning activity in Week 4 – students work on their case study topic individually and share with peer groups for feedback Identifies causes (roots) and effects (branches and leaves) of a given problem (tree)	Students apply critical thinking to evaluate root causes of a problem and effects	A short exercise added in 2025 to support student inquiry about the scales of problems and solutions
Social Entrepreneurship Bootcamp Lecture	Week 6 discussion-based lecture on key components of Social Entrepreneurship	Students deepen their familiarity and working knowledge of key social entrepreneurship methods	Scaffolding and revisiting of several social entrepreneurship topics presented in Week 1 during this discussion-based lecture Topics: *Negotiations, sustainable opportunity recognition & evaluation, market segmentation, Lean Startup Model revisited, Human-Centered Design revisited, Beneficiary experiences, analyzing competition*
Negotiation Strategy	Discussion based lecture in Week 6 ‘Social Entrepreneurship Bootcamp’	Students consider stakeholder/beneficiary experiences before and during negotiations to develop an effective, collaborative strategy	This content was included in the 2024 course with a guest speaker and in 2025 was included in a larger social entrepreneurship lecture by course organizer
Sustainable Ideation	Active learning activity in Week 6 ‘Social Entrepreneurship’ Bootcamp’	Students begin merging concepts in sustainability with social entrepreneurship to understand the importance of interdisciplinarity in solution building and in food systems	A framework created by course organizer asking key sociopolitical questions about a proposed initiative and evaluating each question across economic, environmental and social categories of sustainability
Market Segmentation	Active learning activity in Week 6 ‘Social Entrepreneurship’ Bootcamp’	Students understand the business strategy of market segmentation and can apply this principle to a food system topic	By segmenting possible target markets of an initiative, one can advertise, market, and better address the needs of that specified group of people (beneficiaries), while creating a strategy to expand success across other segments to reach more people
Logic Model Workshop	Lecture and active learning activity in Week 11	Students conceptually understand logic models and theory of change. They apply this knowledge through completion of a logic model specific to their case study topic	This exercise and component of the case study project is the most high-touch aspect of the course for the instructor – as each student needs specific feedback dedicated to the intricacies of their problem, solution, and logic model. Students often reflect upon this as an integral learning experience in the course.
Pitch Workshop	Lecture, videos, and framework for successful pitches discussed in Week 11	Students understand components of compelling pitches and the different formats for different audiences. Students apply this by preparing and delivering a pitch presentation during the final class (Week 15) for their classmates and invited course faculty guests	Students focus on a short pitch format for their case study project presentation to develop compelling, evidence-based storytelling skills and familiarity with a business-based pitch approach.
Winners Take All book discussions	Embedded throughout the class as book chapter discussions. Using journalism, the book criticizes private sector changemaking for lacking public sector accountability	Students evaluate the advantages and limitations of Social Entrepreneurship as a changemaking approach, using real-life examples and their own experiences applying the methods	This book’s journalistic accounts are used as a tool to assess social entrepreneurship’s limitations, helping students understand when and how these methods are relevant within food system and one health work, and under a changing social and political climate

Specific learning objectives and other comments are listed with each method to provide additional context on application of these frameworks in the learning environment.

## 3 Results

### 3.1 Changes in students’ self-assessed topic-based knowledge

Students’ self-assessed knowledge scores for all six key topics (such as food systems, sustainability, social entrepreneurship, One Health, global food security, and impact assessment) improved significantly (*p* < 0.001) throughout the course, with large effect sizes, as displayed in Table 4. The median score for food systems increased from 2.5 on the pre-course survey to 4.0 post-course (1.5 points, *p* < 0.001); increased from 3.0 to 4.0 for sustainability (1 point, *p* < 0.001), increased from 2.0 to 4.0 (2 points, *p* < 0.001) for social entrepreneurship, increased from 3.0 to 5.0 for One Health (2 points, *p* < 0.001), increased from 2.0 to 4.0 for global food security (2 points, *p* < 0.001), and increased from 2.0 to 4.0 for impact assessment (2 points, *p* < 0.001). The Wilcoxon Signed Rank tests and effect size calculations were performed to assess the change in self-assessed knowledge reported from pre- and post-course surveys, and all topics (such as food systems, sustainability, social entrepreneurship, One Health, global food security, and impact assessment) were significant at *p* < 0.001 with large effect sizes, displayed in Supplementary Table 4. To understand whether changes in students’ self-assessed knowledge were constant across course year, students’ scores were stratified by year in Supplementary Table 7, showing that regardless of whether students took the course in 2024 or 2025, each of the six key topics showed significant improvements in self-assessed knowledge with large effect sizes.

**Table 4:** Descriptive statistics for pre- and post-course self-assessed knowledge scores when asked “How would you quantify your knowledge for the following topics from 1 (none at all) to 5 (a great deal).”

Combined 2024–2025	Pre/Post	*N*	Mean	Median	Std. deviation	Min.	Max.	*p*-value	Effect size
Food systems	Pre	20	2.60	2.5	1.095	1	5	<0.001*	0.75
	Post	20	3.95	4.0	0.686	3	5		
Sustainability	Pre	20	2.60	3.0	0.598	2	4	<0.001*	0.85
	Post	20	4.0	4.0	0.649	3	5		
Social entrepreneurship	Pre	20	1.9	2.0	0.788	1	3	<0.001*	0.86
	Post	20	3.80	4.0	0.696	2	5		
One health	Pre	20	3.15	3.0	1.04	1	5	<0.001*	0.80
	Post	20	4.50	5.0	0.607	3	5		
Global food security	Pre	20	2.35	2.0	0.745	1	4	<0.001*	0.87
	Post	20	4.20	4.0	0.696	3	5		
Impact assessment	Pre	20	1.8	2.0	0.834	1	4	<0.001*	0.89
	Post	20	3.95	4.0	0.887	2	5		

To understand the magnitude of change in students’ self-assessed knowledge, a composite score was created for each topic by subtracting each students’ pre-course response from the post-course response. Descriptive statistics of the knowledge composite score are shown in Table 5. The topics with the highest increases in student learning were Impact Assessment (median = 2-point increase, mean = 2.15-point increase), Social Entrepreneurship (median = 2-point increase, mean = 1.9-point increase), and Global Food Security (median = 2-point increase, mean = 1.85-point increase). Food systems (median = 1.5-point increase, mean = 1.35-point increase), Sustainability (median = 1 point increase, mean = 1.4-point increase), and One Health (median 1-point increase, mean = 1.35-point increase) also demonstrated increased student knowledge through the semester.

**Table 5:** Descriptive statistics of knowledge composite scores (composite score = post-survey score–pre-survey score).

	Knowledge composite score					
	Food system	Sustainability	Social entrepreneurship	One health	Global food security	Impact assessment
N	20	20	20	20	20	20
Mean	1.35	1.40	1.90	1.35	1.85	2.15
Median	1.50	1.00	2.00	1.00	2.00	2.00
Std. deviation	1.09	0.75	1.02	0.99	0.88	0.99
Minimum	0.00	0.00	0.00	0.00	0.00	1.00
Maximum	3.00	3.00	4.00	3.00	3.00	4.00

### 3.2 Changes in students’ perceived importance to Global Health

Additionally, students were asked how important they felt those six key topics (such as food systems, sustainability, social entrepreneurship, One Health, global food security, and impact assessment) were to global health, defined as health equity for all, Table 6. While all categories were ranked important in both pre- and post-course surveys, in this exploratory analysis, only the perceived importance of food systems to global health showed significant change (*p* = 0.011) with strong effect size (*r* = 0.57) over the course. The Wilcoxon Signed Rank tests and effect size calculations were performed to assess the change in students’ perceived importance of each topic to global health from pre- and post-course surveys, shown in Supplementary Table 5. As students’ perception of these topics’ importance to global health could be related and not necessarily independent, a Bonferroni correction was performed to assess multiplicity. Using the formula *α*_adjusted_ = α/number of tests, an adjusted *p*-value of 0.0083 (0.05/6 tests) was used to assess significance. In this case, none of the six key topics showed significant change in perceived importance when considered as interrelated.

**Table 6:** Descriptive statistics for pre- and post-course “Importance to Global Health” when asked “How Important do you feel each of these topics are to Global Health (health equity for all) from 1 (not at all important) to 5 (extremely important).”

Combined 2024–2025	Pre/Post	*N*	Mean	Median	Std. deviation	Min.	Maximum	*P*-value	Effect size
Food systems	Pre	20	4.60	5.0	0.598	3	5	0.011*	0.57
	Post	20	5.0	5.0	0	5	5		
Sustainability	Pre	20	4.65	5.0	0.489	4	5	0.059	0.42
	Post	20	4.9	5.0	0.308	4	5		
Social entrepreneurship	Pre	20	3.50	3.0	0.827	2	5	0.509	0.15
	Post	20	3.65	3.50	0.875	2	5		
One health	Pre	20	4.60	5.0	0.598	3	5	0.059	0.42
	Post	20	4.85	5.0	0.366	4	5		
Global food security	Pre	20	4.90	5.0	0.308	4	5	0.564	0.13
	Post	20	4.95	5.0	0.224	4	5		
Impact assessment	Pre	20	3.85	4.0	0.813	2	5	1.00	0.0
	Post	20	3.85	4.0	0.933	2	5		

To understand the magnitude of change in students perceived importance of each topic to global health during the course, a composite score was created for each topic (such as food systems, sustainability, social entrepreneurship, One Health, global food security, and impact assessment). Across each topic, the composite score median was 0; however, most categories had slight positive composite scores means, displayed in Supplementary Table 6.

### 3.3 Changes in students’ understanding of social entrepreneurship

In both the pre- and post-course survey, students were asked to define Social Entrepreneurship. Pre-course survey results showed the majority of students did not know what social entrepreneurship was before the course (11/20 students answered no across both years; 4/11 students answered no in 2024, and 7/9 students answered no in 2025). When students answered no to the question “Do you know what social entrepreneurship is?” on the pre-course survey, they were not required to define the term.

Using a scoring rubric, each definition was scored from 0–3. The rubric evaluation criteria are provided in Supplementary Table 3. Inter-rater reliability assessment of the rubric was performed with a colleague independent of the study and showed 84% alignment in definition scores. A composite score for pre- and post-course definition changes was created. Student definitions of social entrepreneurship improved conceptually as measured by a rubric (median = 2-point increase, mean = 1.7-point increase), and this increase was statistically significant (*p* < 0.001) with a large effect size (*r* = 0.81), as shown in Table 7.

**Table 7:** Descriptive statistics, the Wilcoxon signed rank test, and effect size for a composite score measuring change in pre- and post-course rubric-graded definitions of social entrepreneurship (Composite Score = post-course rubric grade – pre-course rubric grade).

Descriptive statistics	
*N*	20
Mean	1.70
Median	2.00
Standard deviation	1.22
Minimum	−1.00
Maximum	3.00
Wilcoxon signed rank test	
Test statistic	167.00
Standard error	22.627
Standardized test statistic	3.602
Asymptotic sig. (2-sided test)	<0.001*
Effect size (r)	0.805

### 3.4 Changes in students’ learning, attitudes, and Behaviors

In the post-course survey, students were asked to reflect on how course activities impacted their learning. Students rated all activities as having a positive impact on their learning, displayed in Table 8. The pitch presentation and case study project were ranked equally as having the greatest impact on students’ learning (median = 5 and mean = 4.45), followed by logic model creation (median = 4.5 and mean = 4.35), FAIR Farms Gambia social entrepreneurial examples (median = 4 and mean = 4.05), the land sparing and land sharing journal club (mean = 4 and median = 3.89), Winners Take All discussions (median = 4 and mean = 3.65), and in-class social entrepreneurship exercises (median = 3 and mean = 3.3).

**Table 8:** Descriptive statistics on the post-course survey question “Rank how the following course activities impacted your learning from 1 (not useful at all) to 5 (extremely useful).”

	Social entrepreneurship exercises	FAIR Farms Gambia and Gambia Goat dairy as social entrepreneurial examples	Winners take all discussions	Land sharing/Sparing Journal club	Pitch presentation	Logic model creation	Entire case study project
*N*	20	20	20	19	20	20	20
Mean	3.30	4.05	3.65	3.89	4.45	4.35	4.45
Median	3.00	4.00	4.00	4.00	5.00	4.50	5.00
Std. deviation	0.92	0.89	0.93	0.94	0.69	0.75	0.69
Minimum	2	2	2	2	3	3	3
Maximum	5	5	5	5	5	5	5

Lastly, on the post-survey, students were asked how likely they were to do a series of activities to help understand attitudes and behaviors following participation in the course (Table 9). Students responded affirmatively, indicating they were likely to do all listed activities, in ranked order: recommend the course organizer as an instructor (median = 5 and mean = 4.8), recommend studying food systems as part of your degree program (median = 5 and mean = 4.45), recommend this course to others (median = 4.0 and mean = 4.4), recommend social entrepreneurship as a tool for climate action (median = 4, mean = 3.8), recommend the book Winners Take All (median = 4 and mean = 3.8), and use social entrepreneurship in your future endeavors (median = 3.5 and mean = 3.5).

**Table 9:** Descriptive Statistics for the post-course survey question “How Likely are you to ___ from 1 (extremely unlikely) to 5 (very likely).”

	Use social entrepreneurship in your future endeavors?	Recommend this course to others?	Recommend the book winners Take All?	Recommend social entrepreneurship as a tool for climate action?	Recommend studying food systems as part of your degree program?	Recommend [course organizer]?
*N*	20	20	20	20	20	20
Mean	3.50	4.40	3.80	3.80	4.45	4.80
Median	3.50	4.00	4.00	4.00	5.00	5.00
Std. Deviation	0.69	0.60	1.01	1.06	0.83	0.41
Minimum	2	3	2	2	2	4
Maximum	5	5	5	5	5	5

## 4 Discussion

Students self-assessed knowledge increased significantly across all six topics after participating in the course (Table 4), with the largest increases in knowledge in impact assessment, social entrepreneurship, and global food security, all of which showed 2-point increases in composite median scores (Table 5). All topics showed at least a 1-point increase in composite median score, indicating student learning across all topics, with consistent findings across both course years (Supplementary Table 7).

When asked to consider these topics’ importance to global health, an exploratory analysis showed students’ perceived importance changed significantly for food systems through the course (Table 6), while a Bonferonni correction for multiplicity effects within the six factors reduces the *p*-value of significance to 0.0083, making this finding non-significant. Nonetheless, many topics were ranked highly important to global health by students at the start of the course, with food systems, sustainability, One Health, and Global Food Security all having pre- and post-course medians of 5, the highest score. For all six topics, composite score medians did not change (median = 0), while the mean scores increased slightly for all topics except impact assessment (Supplementary Table 6). While all topics were rated as “important” to global health, social entrepreneurship (post-course median = 3.5, post-course mean = 3.65) and impact assessments (post-course median = 4, mean = 3.85) scored lowest of the six topics, but still ranked as somewhat important. The framing of the survey question–*inquiring about importance to global health rather than general importance or importance to One Health*–could contribute to the relatively lower rank for these two topics. It is possible student learning was not yet at an “expert or mastery” level, so to speak, to identify the links between these two concepts in practice and global health outcomes. Relatedly, the survey used the term “impact assessment” while students learned about “logic models” in the course as a universal tool for impact assessment, so terminology differences could also contribute to the lower perceived importance. However, these two topics showed the largest increases in student knowledge gained through the course.

The pre/post composite score of rubric-graded social entrepreneurship definitions showed significant improvement in knowledge of this concept through the course. A correct definition of social entrepreneurship on the rubric included both concepts of “business approach/methodology for social change” and “blending social impact and financial revenue.” The composite score median was a 2-point increase through the course, significant at *p* < 0.001 with a large effect size of 0.805 (Table 7). Interestingly, when grading students’ definitions, 6 of 20 post-course definitions (30%) included the need for “stakeholder,” “community,” “target population,” or “beneficiary” participation in social entrepreneurship. While this concept was not included in the grading rubric, it was a focus of many class activities. Community co-creation was a theme in the social entrepreneurship examples from FAIR Farms Gambia, a demonstration and research farm in The Gambia founded through a participatory process merging social entrepreneurship, One Health, and sustainable agriculture, and the focus of the course instructor’s research. Educational studies have shown that intentionally exposing students to real examples from community partners is a useful teaching strategy to help them understand community engagement and collaboration (40). Similarly, embedding storytelling pedagogy in which students hear real organizational stories, can help foster emotionally resonant classrooms and help students understand complex realities like policy and co-creation (41). As such, emphasis on real-world application of social entrepreneurship through in-class examples with the organization FAIR Farms Gambia could influence students’ critical thinking on the concept. Relatedly, students’ focusing on co-creation could be an effect of “hidden curriculum” – learning both what is explicitly taught and what is implicitly emphasized in the classroom – and as such, students may have favorably viewed methods used in the course instructor’s own work (42, 43). Nevertheless, it was interesting to see this concept become a focus for many students’ post-course social entrepreneurship definitions.

Running a course is a process of continual improvement, with course surveys as a helpful tool. Students scored all course activities as moderately useful (median = 3) or above, with pitch presentation and case study project ranked equally highest, followed in order by logic model creation, FAIR Farms Gambia social entrepreneurial examples, land sparing/sharing journal club, Winners Take All discussions, and in-class social entrepreneurship exercises (Table 8). While the classroom social entrepreneurship learning exercises ranked lowest, the applied aspects of social entrepreneurship–creating a pitch, case study report, and logic model–ranked highest. This aligns with other educational studies that show students perceive pitch presentations and case study projects as satisfactory learning activities (44, 45), along with engineering education evidence from the late 1990’s that utilized case study projects to promote authentic learning (46). Taken together, project-based learning strategies–which can include pitch presentations and case study projects–show consistent positive impacts on student learning outcomes, academic achievement, thinking skills, and affective attitudes, as students critically explore real-world issues by integrating different disciplines, perspectives, and realities through classroom design (47, 48). This can be a possible reason why students in this course ranked applied course activities highly.

“How likely are you to” questions were asked to understand possible student attitudes. These questions showed students were “very likely” to recommend studying food systems in their degree program and “likely” to recommend social entrepreneurship as a tool for climate action and to recommend the course to others (Table 9). Their intended likelihood of using social entrepreneurship, with both a median and mean of 3.5, fell somewhere between the Likert category of “neutral” and “likely,” and can be interpreted as a slightly positive response. Students’ likelihood to recommend social entrepreneurship as a tool for climate action is particularly interesting considering that over half of students did not know what social entrepreneurship was before taking the course.

Social entrepreneurship is one of many systems-based approaches for solution building. As the first exposure to social entrepreneurship for most students, an overall positive likelihood of using the approach in the future is favorable. The course explored the downsides of social entrepreneurship alongside its advantages, by reading the book “Winners Take All” (28). This journalistic account provides examples of how modern entrepreneurship, as a private sector approach, avoids public accountability often embedded in public sector processes, and in some instances, leads to substantial community harms and accumulations of wealth (28). While inclusion of this text was intentional to help students understand, critique, and even shape the future of the approaches we study, this could also lead students to rank the likelihood of their use of social entrepreneurship less favorably due to greater familiarity with the challenges of the approach.

This study has several limitations due to its retrospective and exploratory design. One limitation of the study’s retrospective design is that it draws conclusions from a survey designed to provide feedback on a course and not designed to specifically evaluate One Health learnings. Knowledge gains across the six topics were self-assessed by each student, and as such, were potentially influenced by students’ own perceptions. However, improvements in students’ rubric graded definitions of social entrepreneurship provide more objective evidence of knowledge gained through participation in the course.

With all course surveys, there is potential pressure on students to respond positively, creating social-desirability bias. Students’ perceptions of the course instructor on the survey were high, indicating that social-desirability and instructor-related response biases could have played a role in students’ responses. To mitigate these biases, pre-course surveys were administered before the start of the first class, while post-course surveys were completed individually by students on their own time with guaranteed anonymity. This study is also limited in size, assessing one course with 20 student participants over 2 years, and could be strengthened by evaluating more student learning outcomes and additional One Health curricula. Differences in guest lecturers between the 2024 and 2025 classes could also influence results, though stratified self-assessed knowledge ratings by students for each year showed relatively consistent gains in self-assessed knowledge (Supplementary Table 7). More exploration of the generalizability of the usefulness of social entrepreneurship in One Health curricula and effective educational modalities for operationalization are needed. Despite these limitations, by analyzing existing matched pre- and post-course survey data, this study provides practical insights into student learning through a One Health course.

## 5 Conclusion

Exploratory analysis of student pre- and post-course survey data from a One Health and Global Food Security course that integrated social entrepreneurship showed significant increases in students’ self-assessed topic knowledge and understandings of social entrepreneurship, along with positive attitudes towards the usefulness of the approach. Students’ self-assessed knowledge of six key topics (such as food systems, sustainability, social entrepreneurship, one health, global food security, and impact assessment) increased significantly. Students rated several aspects of applied social entrepreneurship (such as pitch presentation and case study project) as highly impactful for their learning, and their understanding of social entrepreneurship, as evaluated by rubric-graded definitions of the term, improved significantly over the course. Despite most students not knowing the concept before this course, the majority indicated they were likely to use social entrepreneurship in their future endeavors and recommend social entrepreneurship as a tool for climate action. This significant change over a 15-week course suggests that social entrepreneurship may be a relevant approach for One Health instruction.

This research provides an example of utilizing creative One Health course structures and integrating social entrepreneurship as a systems-thinking approach to create statistically significant knowledge gains and attitudinal changes. In the future, additional studies are needed to explore efficacy of other One Health curriculums and approaches to integrating social entrepreneurship in order to help understand the most effective pedagogy for practical instruction of these concepts. Larger numbers of students with wider experiences and backgrounds may also be critical for understanding best practices for One Health instruction. As the field changes and expands, future studies can provide evidence on the operationalization of One Health and the efficacy of educational programs.

## Data Availability

The raw data supporting the conclusions of this article will be made available by the authors, without undue reservation.
