# Supplementary material for: Applying social entrepreneurship to One Health and climate education: a retrospective analysis of pre- and post-course survey data for a One Health and Global Food Security course
Source: Front Vet Sci. 2026 Jun 3;13:1828852. doi: 10.3389/fvets.2026.1828852 (PMC13272292; doi:10.3389/fvets.2026.1828852)
Supplement: Supplementary file 1 [file Table_1.docx]

Supplementary Material

# Supplementary Data

**Supplementary Table 1:** Pre- and Post-Course Survey Questionnaire and Likert Scales

| **Pre/Post Question** | **Survey Question** |
| --- | --- |
| Pre & Post | Student ID Number – *transformed to de-identified participant ID number to match pre and post survey results* |
| Pre-course only | Do you know what social entrepreneurship is?  *If answer is no, student is not required to define the term* |
| Pre & Post | How do you define “social entrepreneurship?” |
| Pre & Post | How would you qualify your knowledge of the following subject areas?   - Food systems - Sustainability - Social entrepreneurship - One Health - Global food security - Impact assessment   **All terms defined on survey as indicated in Supplementary Table __*  *Scale: 1 (none at all), 2 (a little), 3 (a moderate amount), 4 (a lot), 5 (a great deal)* |
| Pre & Post | How important do you feel each of these topics are to global health (health equity for all people)?   - Food systems - Sustainability - Social entrepreneurship - One Health - Global food security - Impact assessment   *Scale: 1 (not at all important), 2 (slightly important), 3 (moderately important), 4 (very important), 5 (extremely important)* |
| Post-course only | Rank how the following course activities impacted your learning:   - Social entrepreneurship exercises - FAIR Farms Gambia & Gambia Goat Dairy as social entrepreneurial examples - Winners Take All book discussions - Land Sharing / Land Sparing Journal Club - Pitch presentations - Logic model creation - Entire case study project   *Scale: 1 (not at all useful), 2 (slightly useful), 3 (moderately useful), 4 (very useful), 5 (extremely useful)* |
| Post-course only | How likely are you to…?   - Use social entrepreneurship in your future endeavors?; - Recommend this course to others? - Recommend the book Winners Take All? - Recommend social entrepreneurship as a tool for climate action? - Recommend studying food systems as part of your degree program? - Recommend [the course instructor]?   *Scale:1 (extremely unlikely), 2 (unlikely), 3 (neutral), 4 (likely), 5 (very likely)* |

**Supplementary Table 2:** Definitions of Key Terms listed on Pre- and Post-Course Surveys

| **Term** | **Definition** |
| --- | --- |
| Food Systems | how food moves from farm to fork, encompassing production, processing, marketing, distribution, and consumption of food |
| Sustainability | social, economic, environmental and socio-political and geo-political factors influencing societal ability to meet one generations' needs without compromising the ability for future generations to meet their own needs |
| Social Entrepreneurship | systems thinking and problem-solving methodologies creating new social business models by blending social impact with profit |
| One Health | the interconnectivity of human, animal, and environmental health. Both an approach to problem solving and a goal for holistic health outcomes |
| Global Food Security | When all people, at all times, have physical, social and economic access to sufficient, safe and nutritious food that meets their dietary needs and food preferences for an active and healthy life |
| Impact Assessment | A structured process to evaluate effectiveness (outputs, outcomes, and impact) of organizational activities and programs |
| Global Health | Health equity for all |

**Supplementary Table 3:** Rubric for scoring student definitions of Social Entrepreneurship

| **Score** | **Definition** |
| --- | --- |
| 0 | No pre-course definition given – *student answered NO to the question “Do you know what social entrepreneurship is?”* |
| 1 | Wrong definition (included none of the listed concepts) |
| 2 | Definition includes 1 of the following concepts of “business approach/methodology for social change” OR “blending social impact and financial revenue” |
| 3 | Correct definition includes BOTH of the following concepts of “business approach/methodology for social change” AND “blending social impact and financial revenue” |

**Supplementary Table 4:** Wilcoxon Signed Rank Test and Effect Size for change in self-assessed knowledge of 6 topics throughout the course

|  | **Food Systems** | **Sustainability** | **Social Entrepreneurship** | | **One Health** | **Global Food Security** | **Impact Assessment** | |
| --- | --- | --- | --- | --- | --- | --- | --- | --- |
| Total N | 20 | 20 | 20 | 20 | | 20 | | 20 |
| Test Statistic | 105.000 | 171.000 | 190.000 | 136.000 | | 190.000 | | 210.000 |
| Standard Error | 15.652 | 22.400 | 24.569 | 18.987 | | 24.497 | | 26.339 |
| Standardized Test Statistic | 3.354 | 3.817 | 3.867 | 3.581 | | 3.878 | | 3.986 |
| Asymptotic Sig.(2-sided test) | <.001* | <.001* | <.001* | <.001* | | <.001* | | <.001* |
| R (effect size) | 0.75 | 0.85 | 0.86 | 0.80 | | 0.87 | | 0.89 |

**Supplementary Table 5:** Wilcoxon Signed Rank Test and Effect Size for change in self-assessed importance to global health of 6 topics throughout the course

|  | **Food Systems** | **Sustainability** | **Social Entrepreneurship** | **One Health** | **Global Food Security** | **Impact Assessment** |
| --- | --- | --- | --- | --- | --- | --- |
| Total N | 20 | 20 | 20 | 20 | 20 | 20 |
| Test Statistic | 28.000 | 24.000 | 47.000 | 10.000 | 4.000 | 22.500 |
| Standard Error | 5.534 | 5.292 | 12.124 | 2.646 | 1.732 | 7.794 |
| Standardized Test Statistic | 2.530 | 1.890 | .660 | 1.890 | .577 | .000 |
| Asymptotic Sig.(2-sided test) | .011* | .059 | .509 | .059 | .564 | 1.000 |
| R (effect size) | 0.57 | 0.42 | 0.15 | 0.42 | 0.13 | 0 |

**Supplementary Table 6:** Descriptive Statistics of Importance to Global Health Composite Scores (Composite Score = Post-Survey Score – Pre-Survey Score)

| **Importance to Global Health Composite Score** | | | | | | |
| --- | --- | --- | --- | --- | --- | --- |
|  | Food System | Sustainability | Social Entrepreneurship | One Health | Global Food Security | Impact Assessment |
| N | 20 | 20 | 20 | 20 | 20 | 20 |
| Mean | .40 | .25 | .15 | .25 | .05 | .00 |
| Median | .00 | .00 | .00 | .00 | .00 | .00 |
| Std. Deviation | .60 | .55 | 1.04 | .55 | .39 | .79 |
| Minimum | .00 | -1.00 | -2.00 | .00 | -1.00 | -2.00 |
| Maximum | 2.00 | 1.00 | 2.00 | 2.00 | 1.00 | 1.00 |

**Supplementary Table 7:** Self-assessed knowledge results stratified by year, 2024 and 2025

| **Year 2024** | Pre/ Post | N | Mean | Median | Std. Deviation | Min. | Maximum | P Value | Effect Size |
| --- | --- | --- | --- | --- | --- | --- | --- | --- | --- |
| Food Systems | Pre | 11 | 2.64 | 3.0 | 1.095 | 1 | 4 | .010* | 0.78 |
|  | Post | 11 | 4.0 | 4.0 | .686 | 3 | 5 |  |  |
| Sustainability | Pre | 11 | 2.64 | 3.0 | .589 | 2 | 3 | .004* | 0.87 |
|  | Post | 11 | 4.0 | 4.0 | .649 | 3 | 5 |  |  |
| Social Entrepreneurship | Pre | 11 | 2.0 | 2.0 | .788 | 1 | 3 | .004* | 0.86 |
|  | Post | 11 | 3.64 | 4.0 | .696 | 2 | 5 |  |  |
| One Health | Pre | 11 | 3.36 | 3.0 | 1.04 | 2 | 5 | .006* | 0.83 |
|  | Post | 11 | 4.55 | 5.0 | .607 | 3 | 5 |  |  |
| Global Food Security | Pre | 11 | 2.36 | 2.0 | .745 | 1 | 4 | .005* | 0.86 |
|  | Post | 11 | 4.18 | 4.0 | .696 | 3 | 5 |  |  |
| Impact Assessment | Pre | 11 | 1.64 | 2.0 | .834 | 1 | 2 | .003* | 0.90 |
|  | Post | 11 | 3.55 | 4.0 | .887 | 2 | 5 |  |  |

| **Year 2025** | Pre/ Post | N | Mean | Median | Std. Deviation | Minimum | Maximum | P Value | Effect Size |
| --- | --- | --- | --- | --- | --- | --- | --- | --- | --- |
| Food Systems | Pre | 9 | 2.56 | 2.0 | 1.13 | 1 | 5 | .026* | .74 |
|  | Post | 9 | 3.89 | 4.0 | .782 | 3 | 5 |  |  |
| Sustainability | Pre | 9 | 2.56 | 2.0 | .726 | 2 | 4 | .010* | 0.86 |
|  | Post | 9 | 4.0 | 4.0 | .707 | 3 | 5 |  |  |
| Social Entrepreneurship | Pre | 9 | 1.78 | 1.0 | .972 | 1 | 3 | .007* | 0.90 |
|  | Post | 9 | 4.0 | 4.0 | .500 | 3 | 5 |  |  |
| One Health | Pre | 9 | 2.89 | 3.0 | 1.269 | 1 | 5 | .017* | 0.80 |
|  | Post | 9 | 4.44 | 4.0 | .527 | 4 | 5 |  |  |
| Global Food Security | Pre | 9 | 2.33 | 2.0 | .707 | 1 | 3 | .007* | 0.90 |
|  | Post | 9 | 4.22 | 4.0 | .833 | 3 | 5 |  |  |
| Impact Assessment | Pre | 9 | 2.0 | 2.0 | 1.118 | 1 | 4 | .017* | 0.80 |
|  | Post | 9 | 4.44 | 5.0 | .762 | 3 | 5 |  |  |
